# A dictionary based informational genome analysis

**DOI:** 10.1186/1471-2164-13-485

**Published:** 2012-09-17

**Authors:** Alberto Castellini, Giuditta Franco, Vincenzo Manca

**Affiliations:** 1 Department of Computer Science, Strada Le Grazie 15, 37134 Verona, Italy

**Keywords:** Comparative genomics, Computational genomics, Genome clustering, Information theory, Sequence analysis

## Abstract

**Background:**

In the post-genomic era several methods of computational genomics are emerging to understand how the whole information is structured within genomes. Literature of last five years accounts for several alignment-free methods, arisen as alternative metrics for dissimilarity of biological sequences. Among the others, recent approaches are based on empirical frequencies of DNA *k*-mers in whole genomes.

**Results:**

Any set of words (factors) occurring in a genome provides a *genomic dictionary*. About sixty genomes were analyzed by means of *informational indexes* based on genomic dictionaries, where a systemic view replaces a local sequence analysis. A software prototype applying a methodology here outlined carried out some computations on genomic data. We computed informational indexes, built the genomic dictionaries with different sizes, along with frequency distributions. The software performed three main tasks: computation of informational indexes, storage of these in a database, index analysis and visualization. The validation was done by investigating genomes of various organisms. A systematic analysis of genomic repeats of several lengths, which is of vivid interest in biology (for example to compute excessively represented functional sequences, such as promoters), was discussed, and suggested a method to define synthetic genetic networks.

**Conclusions:**

We introduced a methodology based on dictionaries, and an efficient motif-finding software application for comparative genomics. This approach could be extended along many investigation lines, namely exported in other contexts of computational genomics, as a basis for discrimination of genomic pathologies.

## Background

Genomes are sequences of nucleotides from hundreds to billions of base pairs long. As sequences of symbols they determine dictionaries, that is, formal languages constituted by words occurring in them. They encode the language of life, as dictating the functioning of all the organisms we consider living beings. A main open problem in science is to find a key to understand such an encrypted language, which more or less directly affects the structure and the interaction of all the cellular and multicellular components [1]. It is like having at hand a book, the language of which has still to be deciphered [2,3]. Namely, the international long-term project ENCODE [4] is searching for encyclopedias, lexicons, catalogs, of DNA biochemically annotated elements in human genome.

Working on genomic dictionaries requires the elaboration of enormous moles of data. As an example, the dictionary of all the substrings of length 18 occurring in *Drosophila melanogaster*’s genome has more than 116 millions of words, which require, only to be stored, non-trivial implementations of *ad hoc* procedures. To the best of our knowledge, exhaustive studies on collections of *k*-mers were carried out for values of *k* which do not exceed 13 (see for example [5-8]).

The starting point of our analysis was the computation of all *k*-mers, with *k *= 6,12,18, of given genomes, listed in Table 1. Some properties of such specific dictionaries and their compared statistics guided our research along lines of development which were in part already present in the literature [9,10], and in part took us towards new topics, which emerged just from the empirical evidence of computed data. An interesting concept in this context is that of *hapax* (a Greek term, meaning “once”, coming from philology, where it is used for denoting a “word said once”). In manuscripts these words are relevant for authorship attribution, in genomes they seem to play essential roles in the genome organization as opposed to *repeat* strings, which instead occur more than once.

**Table 1:** A list of genomes investigated in the paper

**Organism Genome**	**Length (in bp)**	**Genes**	**Type**
*Nanoarchaeum equitans*	490,885	536	Minimal archaeum
*Mycoplasma genitalium*	580,076	476	Minimal bacterium
*Mycoplasma mycoides*	1,211,703	1,016	Venter’s experiment bacterium
*Haemophilus influenzae*	1,830,138	1,717	First sequenced bacterium
*Escherichia coli*	4,639,675	4,685	Bacterium model (K-12)
*Pseudomonas aeruginosa*	6,264,404	5,566	Ubiquitous bacterium
*Saccharomyces cerevisiae*	12,070,898	6,275	Unicellular eukaryote (Yeast)
*Sorangium cellulosum*	13,033,779	9,700	Longest genome bacterium
*Homo sapiens chr. 19*	63,800,000	2,066	Highest gene density H. chromosome
*Caenorhabditis elegans*	100,267,632	19,000	Worm (around 1000 cells)
*Drosophila melanogaster*	129,663,327	14,000	Insect (fruit fly)
*Homo sapiens chr. 1*	247,000,000	3,511	Longest Human chromosome

In Table 1 a list is reported of twelve (out of the sixty we have investigated) genomic sequences, to which we applied the methodology described below. They correspond to genomes of well known organisms, constituting biological models, of relevance in various kinds of genomic analysis. The sequences were downloaded from public websites as FASTA files, and processed by a dedicated Java software that we developed.

In the following basic terminology for genomic dictionaries and multisets, and genomic profiles/distributions, is introduced, along with a simple example focused on a specific DNA sequence. Results are reported in terms of both an analysis of dictionaries of *k*-long hapaxes and repeats, together with the introduction of three related dictionary-based informational indexes, and the definition of *k*-repeat sharing gene networks. Section Discussion is then developed around a phase-transition observed in *k*-dictionaries from *k *= 12 to *k *= 18, and around the structure of genomic information which emerges when dictionary cardinality trends and multplicity-comultiplicity distributions are compared with those of randomly permuted sequences. A description of the software suite developed to perform all our computations is finally presented in section Methods.

### Basic notations

Let us denote by Γ the **genomic alphabet** of four symbols (characters, or letters, associated to nucleotides): Γ={*A*,*T*,*C*,*G*} (then Γ^⋆^, as usual, denotes the set of all possible words over Γ).

A genome *G* is representable by a sequence over Γ, that is, a table assigning a symbol of Γto each position (from 1 to the length of *G*). Symbols are written in a linear order, from left to right, according to the standard writing system of west languages, and to the chemical orientation 5^*′*^−3^*′*^ of DNA molecules. By associating to each symbol of Γthe set of positions where it occurs, *G* may be equivalently identified by four sets of numbers.

All factors (fragments) of a genome *G* are collected in the set *D*(*G*), while we call **k**-**genomic dictionary of*G*** (for some *k *≤ |*G*|), denoted by *D*_*k*_(*G*), the set of all the *k*-long substrings of genome *G*. The **k**-**genomic table***T*_*k*_(*G*), which mathematically corresponds to a *multiset*, is defined by equipping the words of *D*_*k*_(*G*) with their **multiplicities**, that is, the number of their respective occurrences in *G*. Let *α*(*G*) denote the multiplicity of *α* and *pos*_*G*_(*α*) gives the set of positions of *α* in a genome *G* (that is, the positions where the first symbol of *α *is placed). Of course, it holds *α*(*G*) = |*pos*_*G*_(*α*)|. Hence, the table *T*_*k*_(*G*) may be represented by an association of strings to their corresponding multiplicities: *α *↦* α*(*G*), with *α *∈* D*_*k*_(*G*)*.* The sum of all the multiplicities of elements in *D*_*k*_(*G*) is called the *size* of *T*_*k*_(*G*), denoted by |*T*_*k*_(*G*)|, with the same sign for string length and for set cardinality (but the context of use should avoid any confusion). It is easy to realize that:

$$|T_{k}(G)|=|G|-k+1.$$

Word distribution in a genome may be represented along a graphical profile, which measures the number of *k*-words having a given number of occurrences. Words having the same multiplicity in a *k*-genomic table *T*_*k*_(*G*) can be grouped and their number is called **comultiplicity**. As an instance, for the sequence *ATTAGGATCTTAAT*, we have: six 2-words occurring once (i.e., AA, AG, TC, CT, GA, GG), two words occurring twice (i.e., TA, TT), one word (i.e., AT) occurring 3 times, and seven 2-words which do not occur at all.

**Figure 1:**
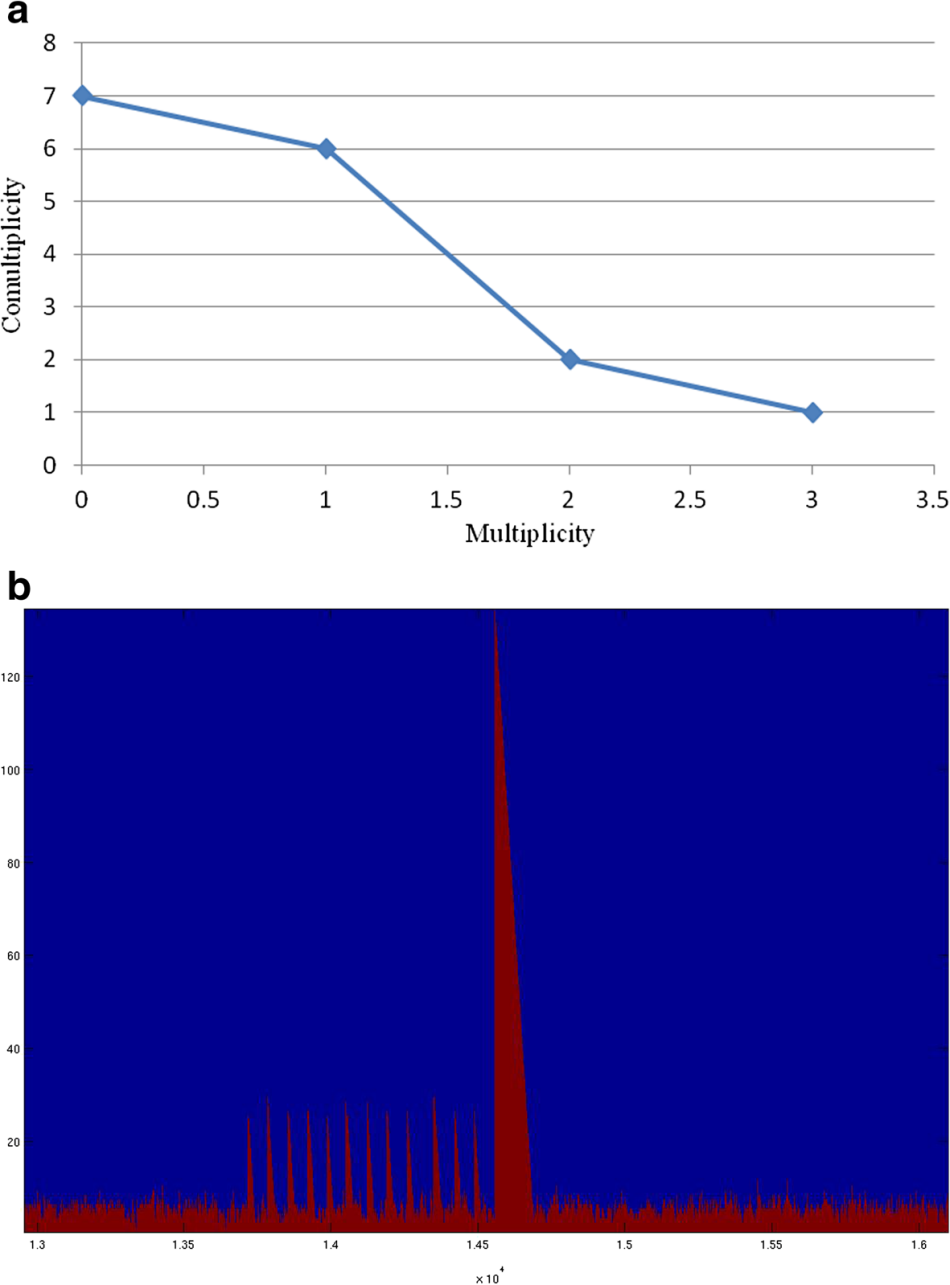
**(a) Multiplicity-comultiplicity 2-distribution of the sequence ATTAGGATCTTAAT.** A simple example of a multiplicity-comultiplicity 2-distribution diagram for the specific sequence *ATTAGGATCTTAAT* is here reported **(b)** Localization of some repeats. A diagram is shown for localization of repeats in the range 1.3 - 1.6 ×10^4^of N. equitans’ genome, where one repeat of 130 occurs, after a few shorter ones (about 30). Positions versus repeat lengths are respectively reported on the axes.

**Figure 2:**
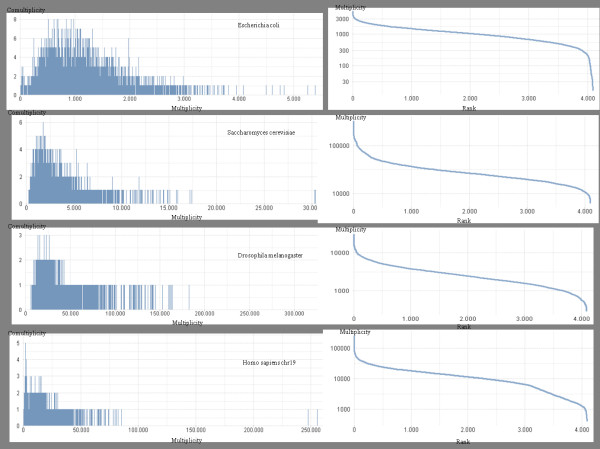
**Multiplicity-comultiplicity and rank-multiplicity distributions.** Some examples of multiplicity-comultiplicity *k*-distributions and Zipf’s curves [11] are reported, related to the 6-genomic dictionary of *Escherichia coli*, *Saccharomyces cervisiae*, *Drosophila melanogaster*, and chromosome 19 of *Homo sapiens* respectively. On the left, we report a given multiplicity on the *x*-axis, and the number of 6-words having that multiplicity on the *y*-axis. On the right, we have the corresponding Zipf’s distributions, where the words of the 6-genomic dictionary are reported on the *x*-axis, according to their decreasing number of occurrences (words with a same number of occurrences are lexicographically ordered), which is on the *y*-axis, in logarithmic form.

If we report 2-words multiplicities on the *x*-axis and their number (comultiplicity) on the *y*-axis, we obtain the chart in Figure 1a. We call such curves **multiplicity-comultiplicityk-distribution** (see Figure 2) of a genome. This kind of charts [5] represents a recent approach in genome analysis, opening new investigation lines about the internal logic underlying genome organizations. The same information may be graphically reported as a rank-multiplicity Zipf map (usually employed to study word frequencies in natural languages [11]). As one may notice by looking at Figure 2, both the middle and final inclination of Zipf’s curves is different for four of our organisms, accounting for the multiplicity range in which we have a major density of strings. In all cases, we have few units with maximal multiplicity, indeed Zipf curves initially slope down steeply.

Several other nice representations of genomic frequencies may be found in the literature, for example by means of images (in [7], distance between images results in a measure of phylogenetic proximity, especially to distinguish eukaryotes from prokaryotes).

## Results

Two important types of factors of genomes are hapaxes and repeats. A **hapax** of a genome *G* is a factor *α* of *G* such that *α*(*G*) = 1. A **repeat** of *G* is a factor *α* of *G* such that *α*(*G*) > 1. Two or more contiguous occurrences of one repeat form a sequence technically called *tandem repeat*, if the repeated sequence is shorter than 10 nucleotides, one has a *minisatellite* or *short tandem repeat*. They describe patterns helpful to determine individual’s inherited traits, namely to determine parentage or genealogical information.

Back to the dictionaries, the set *H*(*G*) of hapaxes of *G* and the set *R*(*G*) of repeats of *G* of course constitute a bipartition of *D*(*G*) (at least one element of Γ is a repeat and *G* is a hapax, therefore *H*(*G*) and *R*(*G*) are nonempty, also disjoint sets, such that their union is *D*(*G*)). We set

$$H_{k}(G)=\Gamma^{k}\cap H(G)\;\;\text{and}\;\;R_{k}(G)=\Gamma^{k}\cap R(G)$$

where ∩ is the set-theoretic intersection.

Therefore, given a genome *G* of length *n*, for any *k *≤* n *we can read it according to the bi-partition of its *k*-genomic dictionaries *H*_*k*_(*G*) and *R*_*k*_(*G*). Size variations of *k*-genomic, *k*-hapax and *k*-repeat dictionaries, for *k *= 1,…,18, are analyzed in the following (see Tables 2, 3, 4 for numerical data), while the size of “forbidden dictionaries” (those composed by “non-appearing” *k*-words, said also “nullomers” [12]), for given genomes, is of course exponentially increasing with *k*.

**Table 2:** **Indexes related to *D***_**6**_**(*G*)**

**Genomic Sequences**	**|*D***_**6**_**|**	***L***_6_	**|*H***_**6**_**|**	**|*R***_**6**_**|**	***HR***_**6**_	
Nanoarchaeum equitans	4,094	0.008	6	4,086	1.468 ×10^***−3***^	
Mycoplasma genitalium	4,082	0.007	35	4,047	8.65 ×10^−3^	
Mycoplasma mycoides	4,076	0.003	39	4,037	9.661 ×10^−3^	
Haemophilus influenzae	4,096	0.002	0	4,096	0	
Escherichia coli	”	0.0009	”	”	”	
⋮	”	⋮	”	”	”	

**Table 3:** **Indexes related to *D***_**12**_**(*G*)**

**Genomic Sequences**	**|*D***_**12**_**|**	***L***_**12**_	**|*H***_**12**_**|**	**|*R***_**12**_**|**	***RD***_**12**_	***H*R**_**12**_	***A*R**_**12**_	
Nanoarchaeum equitans	431,046	0.87	385,146	45,900	0.11	8.39	2.30	
Mycoplasma genitalium	496,194	0.85	435,502	60,692	0.13	7.175	2.38	
Mycoplasma mycoides	646,965	0.53	442,836	204,129	0.32	2.169	3.76	
Haemophilus influenzae	1,495,701	0.81	1,256,043	239,658	0.17	5.240	2.39	
Escherichia coli	3,478,923	0.74	2,675,846	803,077	0.24	3.331	2.44	
Pseudomonas aeruginosa	2,949,852	0.47	1,799, 637	1,150,215	0.39	1.564	3.88	
Saccharomyces cerevisiae	6,597,259	0.54	3,977,392	2,619,867	0.40	1.518	3.08	
Sorangium cellulosum	3,863,399	0.29	1,924,969	1,938,430	0.51	0.993	5.73	
Homo sapiens chr19	10,735,683	0.19	3,359,705	7,375,978	0.69	0.455	6.99	
C. elegans	13,929,915	0.13	3,099,744	10,830,171	0.78	0.286	8.97	
D. melanogaster	15,891,212	0.12	1,632,045	14,259,167	0.9	0.114	8.89	

**Table 4:** **Indexes related to *D***_**18**_**(*G*)**

**Genomic Sequences**	**|*D***_**18**_**|**	***L***_**18**_	**|*H***_**18**_**|**	**|*R***_**18**_**|**	***RD***_**18**_	***H*R**_**18**_	***A*R**_**18**_
Nanoarchaeum equitans	489,465	0.99	488,802	663	0.001	737.25	3.11
Mycoplasma genitalium	569,202	0.98	563,045	6,157	0.01	91.44	2.76
Mycoplasma mycoides	987,645	0.81	913,599	74,046	0.07	12.33	4.025
Haemophilus influenzae	1,795,492	0.98	1,775,531	19,964	0.01	88.93	2.64
Escherichia coli	4,557,590	0.98	4,518,585	39,005	0.008	115.84	3.10
Pseudomonas aeruginosa	6,183,215	0.98	6,117,968	65,247	0.01	93.76	2.24
Saccharomyces cerevisiae	11,499,795	0.95	11,307,098	192,697	0.01	58.67	3.96
Sorangium cellulosum	12,640,960	0.96	12,340,846	300,114	0.02	41.12	2.30
Homo sapiens chr19	41,529,106	0.75	39,256,297	2,272,809	0.05	17.27	6.91
C. elegans	89,444,661	0.89	85,157,627	4,287,034	0.04	19.86	3.52
D. melanogaster	116,446,627	0.90	112,977,046	3,469,581	0.02	32.56	4.45

According to data reported in Table 2, in the first three genomes of the list, |*D*_6_(*G*)| slightly decreases and repetitiveness slightly increases for longer genomes. When the analyzed genomes length exceeds about 1,800,000 base pairs, the decomposition of *D*_6 _in hapaxes and repeats keeps the identical respective cardinalities. All the 6-genomic dictionaries are composed by only repeat words (i.e., they do not contain any hapax).

In Table 3, the number of hapax words |*H*_12 _(*G*)| appears not related to the length of genome G, and neither to the cardinality of *D*_12_(*G*); while the ratio of 12-hapaxes over 12-repeats *HR*_12 _appears roughly decreasing with the genome length. This is due to the fact that 12-repeat words constitute a considerable portion of 12-genomic dictionary, actually a percentage (called *RD*_12_) which increases with the genome length (from 11% to 90%). The average 12-factors repeatability index, in the last column, accounts for the average repeatability of 12-repeats in all the genomes.

In Table 4, cardinality of *D*_18_and *H*_18_increase with the genome length, as expected. As a notable result though, we can see that the 18-repeat-factor ratio *RD*_18_ is firmly fixed (over all the genomes) on a very small portion of the 18-genomic dictionary, mostly ranging from 0.01 to 0.07 (and always less than 1%), independently on the genome length. The 18-hapax-repeat ratio *HR*_18_ does not show a regular behavior with respect to the length, but its values are considerably greater for longer words (according to the data, for *k *= 12 and *k *= 18). The average 18-factor repeatability index does not exhibit the regularity of the average 12-factor repeatability with respect to the genome length, it even shows an exceptionally high value for the chromosome 19 of *H. sapiens*.

It is easy to see that any genomic factor containing a hapax as a substring is an hapax as well. Hence an hapax within the genome may be elongated (by keeping its property to be an hapax) up to reach the genome itself, which is of course an hapax. It is then interesting to evaluate, for each genome *G*: *i)* how |*H*_*k*_(*G*)| varies with *k* (see http://www.cbmc.it/external/Infogenomics3), *ii)* the *k*-hapax positions (that is, how densely hapax words fall in the genetic regions), and *iii)* the shortest length of an hapax. Also, a *k*-similarity between genomes *G* and *G*^*′*^ could be measured by |*H*_*k*_(*G*)∩*H*_*k*_(*G*^*′*^)| (we have some work in progress on the computation of dictionary intersections).

The concepts of hapax and repeat provide a great number of related notions which permit to define important aspects in the analysis of real genomes. In following sections we will discuss numerical data, reported in tables, diagrams, and figures, which include the measure of the ratio between |*H*_*k*_(*G*)| and |*R*_*k*_(*G*)| as a function of *k* (that is, how the number of hapax words of a given length increases or decreases with respect to the number of repeats of that length). We observed a sort of *transition phase* effect in the passage from *D*_12_(*G*) to *D*_18_(*G*), in almost all genomes of Table 1, where a clear inversion appears in the ratio hapax-cardinality/repeat-cardinality.

### Dictionary based indexes

For a genome *G* we may define **k**-**lexicality**, that is, the ratio *L*_*k*_(*G*) = |*D*_*k*_(*G*)|/|*T*_*k*_(*G*)|, which expresses the percentage of distinct *k*-factors of *G* with respect to the all the *k*-factors present in G (in Tables 2, 3, 4, it is clear that the *k*-lexicality increases with the word length *k*, and does not exhibit any regularity with the genome length). Of course, the inverse of this ratio provides an average repeatability of *k*-factors in *G*.

A more refined measure for the **averagek**-**factors repeatability** in *G* may be now given as:

$$AR_{k}(G)=\frac{\left|T_{k}(G)\setminus H_{k}(G)\right|}{\left|R_{k}(G)\right|}$$

where *k*-hapaxes have been excluded by both the *k*-genomic multiset and the *k*-genomic dictionary (the symbol ∖ represents the set-theoretic difference). Index *AR*_*k*_(*G*) counts the proper (average) repeatability of *k*-repeats in genome G (see Tables 3 and 4 for computed numerical values).

Finally, *maximal repeats* of a genome *G* are substrings occurring at least twice and having maximal length. Some numerical indexes related to this concept are *i)* the maximal repeat length *MR*(*G*), *ii)* the number of different maximal repeat sequences, and *iii)* the number of times each maximal subsequence is repeated (see Table 5).

**Table 5:** MR index and MR-repeat distance

**Genomic Sequences**	**MR**	**M*D***_***MR***_**/|G|**
*Nanoarchaeum equitans*	139	96.95%
*Mycoplasma genitalium*	243	0.15 %
*Mycoplasma mycoides*	10,963	0.019 %
*Haemophilus influenzae*	5,563	8.05%
*Escherichia coli*	2,815	0.89 %
*Pseudomonas aeruginosa*	5,304	12.37 %
*Saccharomyces cerevisiae*	8,375	0.07%
*Sorangium cellulosum*	2,720	27.68 %
*Homo sapiens chr19*	2,247	0.02%
*C. elegans*	38,987	0.10 %
*D. melanogaster*	30,892	0.02 %

All genomes turned out to have only one repeat having maximal length (and multiplicity 2), and the distance of the two positions (in proportion to the genome length) is reported in Table 5. They are in most cases relatively very close. Although for *k *= 6,12,18, |*R*_*k*_| increases with the genome length *n*, there is no apparent correlation between *n* and the MR index (in all cases |*R*_*MR*_| = 2).

Any substring of a repeat word is still a repeat, with an own multiplicity along the genome, and inside the repeat word itself. A further index is thus defined over genomes G, called *MR*(*G*) (**maximal repeat length**), as the maximal length of words Γsuch that *γ*(*G*) > 1. An algorithmic way to find it (for our genomes) starts from repeats out of *D*_18_(*G*) (that are less than three a half millions) and checks how much they may be elongated on the genome by keeping their status of repeat words. Data related to the MR index computed over our genomes are reported in Table 5, where the only MR-long repeat of each genome exhibits a non-trivial structure (that is, different than polymers with a same nucleotide or similar patterns), and complex repeats are obtained for many lengths.

The importance of word repeatability is crucial in understanding the information content of texts. A genome analysis in terms of (shortest) hapaxes and (maximal) repeats, providing their relative distribution within the genome, highlights the associative nature of DNA as a container of information [13]. Localization (see Figure 1b) and frequency (see Figure 2) of DNA fragments of specific length is indeed crucial in understanding the information organization of genomes [14].

### Repeat-sharing gene networks

**Figure 3:**
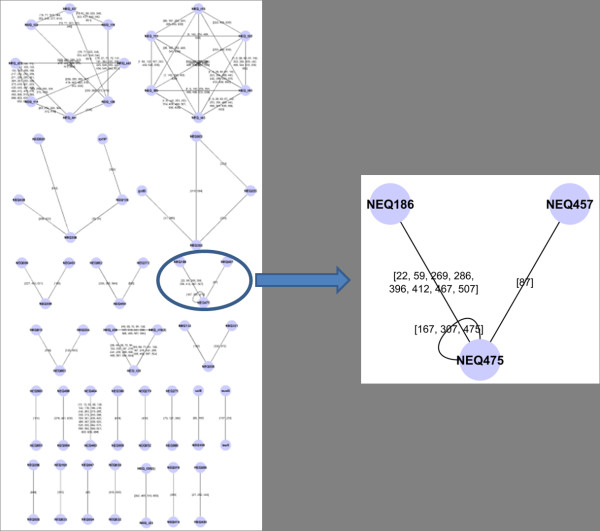
**Repeat sharing gene network of N. equitans.** A subgraph is pointed out of the 18-repeat sharing gene network of Nanoarcheaum equitans, a short genome (see Table 1) which is mostly (93%) formed by genes. As we may notice on the right, the gene NEQ475 is linked with the NEQ186 and NEQ457. It contains at least two occurrences of each of three different repeats, has 8 distinct repeats in common with NEQ186 and only one with NEQ 457.

**Figure 4:**
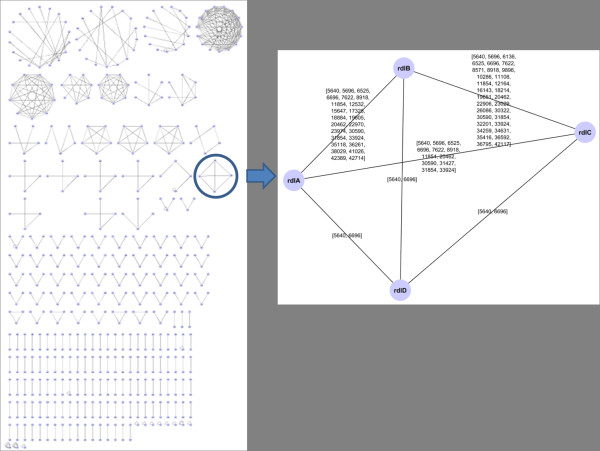
**Repeat sharing gene network of*E. coli*.** A subgraph is pointed out of the 18-repeat sharing gene network of Escherichia coli, whose genome has an high percentage (89%) of genes. Four genes in the figure on the right turn out all connected, by only one repeat in half of the connections, and a quite high number of common repeat in the others.

Once we discovered that the percentage of repeats in dictionaries is “low” (and decreasing with *k*), we focused on studying the positions of 18-repeats along the genome, in order to check if they are more densely present in encoding regions or non-coding ones. This investigation allowed us to design a synthetic gene network in the following way: nodes are genes, and they are connected by an edge if they have at least one common repeat (that is, there exists a repeat which is a proper factor common to the two genes). An interest for this kind of diagram (see examples in Figures 3 and 4) finds a motivation in the hypothetic communication between genes due to competitions for short endogenous RNA sequences (around 20 bases long) proposed in [15].

We have work in progress to investigate these *k*-parametrized labeled gene networks by standard methods of graph theory and network analysis. Gene nodes with higher degrees turned out to be actually involved in important long genetic pathways, and for specific values of *k*, between 16 and 18, drastic changes may be observed in the network conformation, while emerging several clusters of genes. However, this is out of the scope of this work, even if it will be a natural extension of it.

## Discussion

In this session we would like to specifically discuss the computational results reported in all the tables, and the importance of reading a genome by its mutliplicity-comultiplicity *k*-distribution. In both cases internal structural properties of genomes emerge which highlight regularity indicators, based on the number and distribution of repeats.

For all our genomes of Table 1, listed according to an increasing genome length order, we report in Tables 2, 3, and 4 numerical data related to the computation of *D*_*k*_(*G*),*H*_*k*_(*G*),*R*_*k*_(*G*) for *k *= 6, 12, and 18, respectively^a^.

A peculiar phenomenon regarding hapax statistical distribution may be observed passing from the 12- to the 18-genomic dictionary (see Tables 3 and 4). For all the genomes, by enlarging the *k* value, the number of hapax increases, even relatively to the number of repeats (roughly speaking, “most of the 12-words are repeats while most of 18-words are hapax”). Indeed, by computing $HR_{k}=\frac{\left|H_{k}\right|}{\left|R_{k}\right|}$ for *k *= 12,18, we see that repeatability generally increases with genome length for *k *= 6,12, while this regularity disappears for *k *= 18.

**Figure 5:**
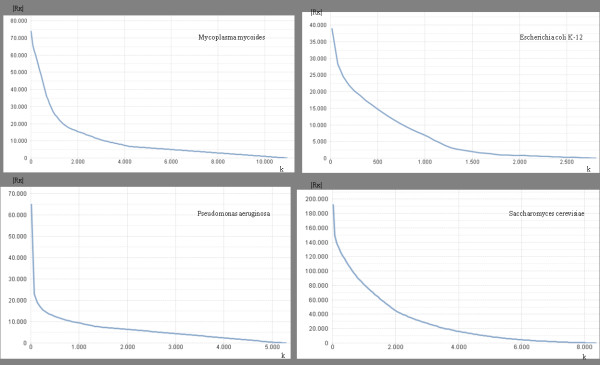
**Length-cardinality repeat distributions.** In this figure four examples are reported, related to the MR computations of *Mycoplasma mycoides, Escherichia coli, Pseudomonas aeruginosa, and Sorangium cellulosum*. Here we observe that |*R*_*k*_| has an exponential decay with the word length *k*. Moreover, very long repeat words were found for any of the genomes we analyzed.

More interestingly, the (relative) amount of hapaxes increases by some orders of magnitude with *k* passing from 12 to 18. Based on this observation coming from computational experiments, one could suppose that by increasing the word size, genomic dictionaries composed of only hapaxes may be computed (which would have been good news for genome reconstruction algorithms [16,17]). This intuition though has been invalidated by further computations (see Table 2). In fact, repeats having length of several thousands have been found within each of our genomes (see for example Figure 5, and the website http://www.cbmc.it/external/Infogenomics3), and 12→18 represents a sort of phase transition from scarce to abundant hapax/repeat distribution. This phenomenon would surely deserve a more detailed and generalized analysis.

### Random vs real genomes

We have carried out a systematic study of repeat distribution, of real and randomly permuted genomes (that are, random sequences having the same nucleotide frequencies of the original genome), in order to get new information on the structure of such relevant motifs [14].

**Figure 6:**
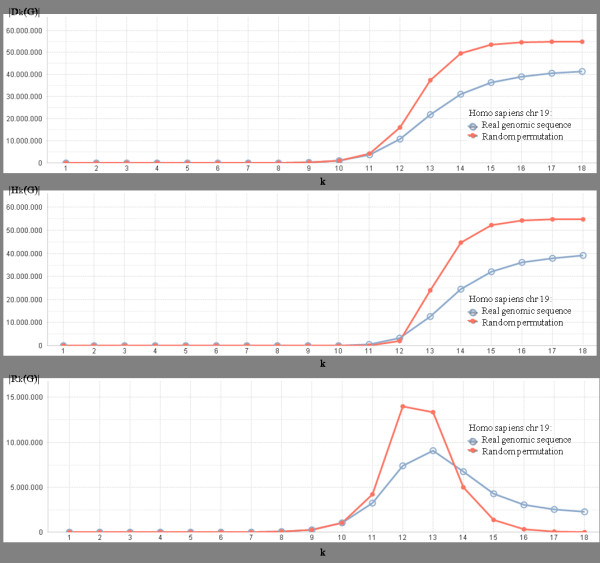
**Cardinality trends of*|D***_**k**_***(G)|*(chart on top),*|H***_**k**_***(G)|*(second chart), and*|R***_**k**_***(G)| *(bottom chart), for G being the*Homo sapiens (chromosome 19)*, and for*k = 1,…,18*.** Blue lines (big dots) represent dictionary trends of real genomic sequence, and red lines (small dots) represent dictionary trends for a random permutation of the same genomic sequence. First chart: number of genomic *k*-words; Second chart: number of hapax *k*-words; Third chart: number of repeat *k*-words.

We produced some diagrams showing how the number of genomic, hapax, and repeat words of a given length varies with respect to the length (see website http://www.cbmc.it/external/Infogenomics3), and a common remarkable finding is the similar shapes of the curves, where the transition aforementioned occurs. Cardinality trends of sets *D*_*k*_(*G*) (dictionary words), *R*_*k*_(*G*) (repeat words), and *H*_*k*_(*G*) (hapax words), for *k *= 1,…,18 are compared for genomes and their random permutations, and specifically for Human chromosome, a greater difference between random and non-random situation may be clearly observed (see Figure 6).

If we compare the dictionaries of the genome with those of its random permutation (in Figure 6, respectively, big blue versus small red dots), we find quite similar curves. However, even when diagrams follow the same general trends, specific characters of these curves correspond to features which are typical of the single genomes [18]. In general, random values are always considerably greater than non-random values, for both hapax and whole dictionaries, while the opposite appears for repeats, before and after the distribution peaks.

All the data were confirmed along with several random permutations. However, apart of the comparison with permuted sequences, we would like to observe the shape of |*R*_*k*_| in itself. Only in a limited range of values for *k*, *R*_*k*_has a significant size, and such a range is [7,17] for all the analyzed genomes, with a pick around the value *k*=10, while both shifting towards the values 11, 12 for the pick, with the increasing of genome length.

Multiplicity-comultiplicity charts have been computed for all the genomes as well, by means of an application of the software described in the Methods section. displays some of them for 6-words of four organisms: *Escherichia coli*, *Saccharomyces cervisiae*, *Drosophila melanogaster* and *Homo sapiens (chromosome 19)*. Blue bars are related to real genome sequences and red bars concern random permutations of the same sequences. At a first glance, in real genome distributions (blue bars) we notice a common trend, very similar to a Poisson distribution, with specific peculiarities which characterize each genome. On the other hand, random permutations of genomic sequences have multimodal distributions which depend on base frequencies.

**Figure 7:**
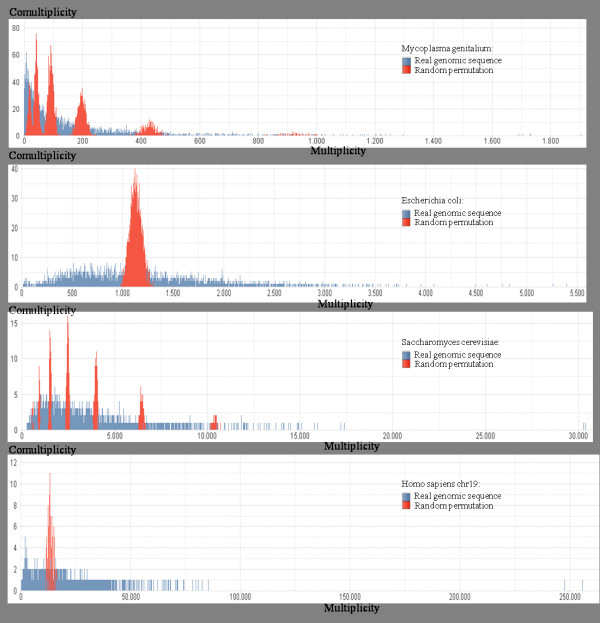
**Multiplicity-comultiplicity 6-distributions for*Escherichia coli*,*Saccharomyces cervisiae*,*Drosophila melanogaster* and*Homo sapiens (chromosome 19)*.** Blue bars represent distributions of real genomic sequences, while red bars represent distributions of random permutations of genomic sequences

We observe that the multplicity-comultiplicity distribution of *Escherichia coli* has multiplicities (*x*-axis) between about 0 and about 5,400, whereas *Drosophila melanogaster* has multiplicities between about 5,000 and about 330,000. On the other hand, the maximum comultiplicity is 8 for *Escherichia coli*, and is 3 for *Drosophila melanogaster* (in Figure 7, see the *y*-axis of the first and the third charts). These parameters are very different even if the “shape” of the genomic sequences in the two charts is quite similar. In order to perform a comprehensive analysis of multplicity-comultiplicity distribution we have dealt with them as probability distributions, and we have computed about 25 statistical indexes which characterize them, such as, maximum, minimum and mean multiplicity, maximum, minimum and mean comultiplicity, standard deviation, kurtosis, skewness, mode, entropy, etc. In [18] these indexes have been successfully employed to classify genomes according to their organism kingdom.

As a conclusion, in Figure 7 we would like to point out that in cases of random permutations of genomes, multimodal shapes may be observed, which depend on the base frequencies of genomes. However, the apparently more ordered concentrations of word multiplicities, around the modes, can be explained by considering that frequencies allow us to classify (and count) words corresponding to the same multiset (Parikh vector equivalent). Consequently, due to the random effect, being the words with the same multiset equally probable, they concentrate around the multiplicity associated to this probability. These distribution differences between randomly permuted genomes and real genomes is another measure of the information content that genomes have with respect to casual sequences.

## Conclusions

Bipartition of a genomic dictionary in hapax and repeat words emphasizes the roots of precise string categories which are related to the functional organization of genomes. The set of 18-repeats in our genomes has a digital size which is a couple of orders smaller than the whole genome, and it seems to have a role of “lexical” coding, that is, a semantics external to the genome. Other elements, with a notably bigger digital size, seem to have a role of addressing, delimiting, coordinating, just like position-identification tags.

The definition, computation, and analysis of well characterized dictionary based genomic indexes have pointed out some phenomena of genomic regularity and specificity. They can highlight our knowledge about the internal logic of genome structure and organization, as well as about evolutional and functional attributes of genomes (as in [18], specifically devoted to genome clustering).

### Future work

There are several lines of development that our research is intended to pursue. We are already working on some of these, mainly focused on the study of intersections among genomic dictionaries. It would be interesting to check the relationship between words recurrent in dictionary intersections and those which are known to be conserved along the evolutive lineages. Another research line concerns the inter-genomic character of hapaxes and repeats. The question is about which hapaxes (respectively repeats) of a given genome occur in other genomes of a certain class by keeping their status of hapax (resp. repeat) when compared to the new context of words.

Finally, we conclude with a fundamental question which points out a novel perspective related to the approach developed in the paper: what is the essence of a genome? For genome functions, two aspects are essential: the presence of some factors and their relative positions. Discovering which factors are essential, the classes related to their roles, and the mechanisms for expressing their relative positions, could provide essential properties of genomes, even without a detailed knowledge of their whole sequence. The approach outlined in this paper could be considered as a first step in the exploration of this perspective.

## Methods

**Figure 8:**
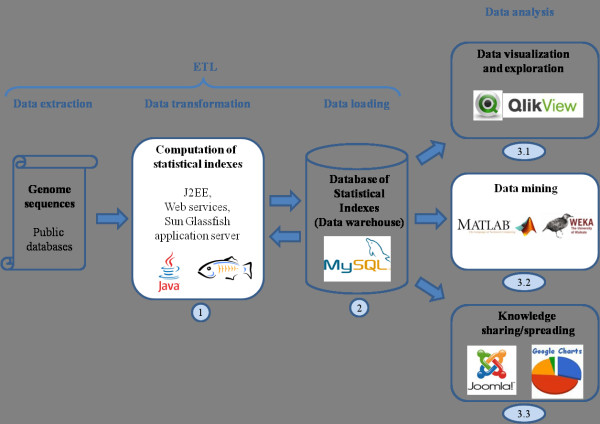
Genome analysis process and software architecture.

The genome analysis described so far requires a rigorous protocol and a sophisticated technological infrastructure in order to be performed systematically. Dictionaries, tables, distributions and related indexes, described so far, need a lot of computational resources to be calculated, and advanced data exploration and visualization tools to be analyzed. We have developed a process (and a related software suite), shown in Figure 8, for informational index generation and analysis. It involves three main phases: *(i)* acquisition of genomic sequences from public databases, *(ii)* computation of informational indexes, which are subsequently stored in a database, *(iii)* visualization, exploration and quantitative analysis of these informational indexes.

Sequences were downloaded as FASTA files from *NCBI genome database*[19], UCSC Genome Bioinformatics website [20] and EMBL-EBI website [21], and they were stored, with their accession numbers and identification data, on our server. About sixty sequences have been analyzed so far, corresponding to genomes of well known organisms, often constituting biological models, of remarkable relevance in the genomic analysis. All classes of Archea, Bacteria, and Eucaryotes^b^ are represented.

The software employed to process genomic sequences and to compute informational indexes is a sophisticated service oriented architecture based on Java web services. The Java EE application model guarantees the scalability, accessibility, and manageability needed by our application. Each index is computed by a specific web service which receives as an input a genomic sequence with some additional parameters, and stores the results in a *MySQL* database, representing the data warehouse of our infrastructure.

Optimized data structures and algorithms were required to perform index computation since huge amount of data had to be processed. The entire application is hosted by a high performance server having 16 processors and 24GB of RAM. Our index database currently contains about 100GB of data, consisting of 300 millions of records. The amount of information generated by web services is sometimes very large (e.g., a 12-genomic dictionary *D*_12_(*G*) could have up to 4^12 ^≈ 16 millions of words) and the storage of this information in databases could require quite a lot of time and specific database setting. The advantage to use web services to compute informational indexes is that they can be called by many kinds of application clients. In this section we have described only a *Java* application client, but web clients or non-Java clients (e.g., *Microsoft.Net* or *Matlab* clients) could be employed as well. Web services guarantee a great interoperability and extensibility to our application.

The visualization and exploration of such an enormous dataset requires specific tools as well. We have adopted a data access solution, called Qlik^*®*^View [22], coming from the world of *Business Intelligence* (where sophisticated elaborations of huge moles of economic and financial data are performed). This tool enables an interactive exploration of large and complex datasets by means of a patented *in-memory associative technology*.

**Figure 9:**
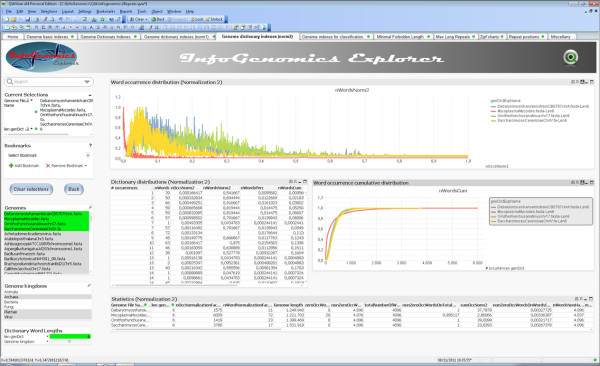
**Visualization and exploration of informational indexes by means of a Qlik**^**®**^**View application called InfoGenomics.** Multiplicity-comultiplicity distributions of four genomic sequences are visualized in the same (central) chart in order to visually compare their profiles. The figure shows a table where number of occurrences and related number of words are listed, and can be selected in order to focus the exploration on specific features. A second chart, placed on the right, shows cumulative distributions, and a table placed on the bottom shows statistical indexes (e.g., mean, standard deviation) related to the distributions.

Figure 9 shows a screenshot of the Qlik^*®*^View application, which has two main sections. A *navigation menu*, on the left, by which the user can select genome sequences, organism kingdoms and dictionary parameters. A *central area* containing visualization elements of genomic indexes, such as tables, charts, lists of words, and diagrams.

Tabs differ only in the central area, where informational indexes are displayed by means of several kinds of graphical objects provided by Qlik^*®*^View. This way to visualize and browse the information is very powerful and enables the user to achieve a deep insight into the genomes. The following list summarizes the functionalities developed so far which contained in the tabs: genome basic indexes (genome identificators, base frequencies, gc-content, etc.); *k*-Dictionaries and Multiplicity-Comultiplicity distributions; normalizations of indexes at the previous item; statistical parameters (e.g., mean, standard deviation, mode, *k*-empirical entropy, etc.) related to Multiplicity-Comultiplicity distributions; dictionary intersections; maximal repeat lengths; dictionary size trends.

## Endnotes

^a^When analyzing downloaded genomes, in some cases we have found a number *num* of *unavoidable words*, defined as those containing IUPAC (variable) symbols, which can assume one of the values A, T, C, G (see http://www.mun.ca/biochem/courses/3107/sym-bols.-html). When they are present in a genome, such as the case of Haemophilus Influenzae, they are eliminated from the computation of all words in the genome, then the *k*-genomic dictionary is built up not from *n*−*k* + 1 genomic *k*-long words, but from the *n*−*k*−*num* + 1 regular words. Specifically, as value of *num* we have found: for *H. influenzae*’s 6/12/18-genomic dictionary, respectively 646, 1,271, 1,877; for *D. melanogaster*’s 6/12/18-genomic dictionary, respectively 1,225,656, 1,226,400, 1,227,144; for *H. sapiens*’ 6/12/18-genomic dictionary, respectively 1,171,155, 1,173,045, 1,174,935.

^b^A most detailed description of these genomes may be found in: http://use-rs.rcn.com/jkimball.ma.ultranet/BiologyPages/G/Genome-Sizes.html.

## Competing interests

The authors declare that they have no competing interests.

## Author’s contributions

This paper is a first step towards a project, called Infogenomics, conceived and designed by the last author VM, who defined an initial kernel of informational indexes, to be investigated and compared on specific genomes. A sophisticated service oriented architecture (SOA) based on Java web services and a Qlik^*®*^View application, has been developed by the first author AC, in order to make possible the high amount of computations necessary for the informational analysis of genomes. All the authors discussed and agreed on the interpretation of experimental results, with a main role of GF in the preparation of the paper. All authors read and approved the final manuscript.

## References

[B1] Gibson DG Creation of a Bacterial Cell Controlled by a Chemically Synthesized Genome Science 2010 329 5987 52 56 10.1126/science.1190719 20488990

[B2] Gibson G Muse SV A Primer of Genome Science 2009 Sinauer Associates Inc

[B3] Percus JK Mathematics of Genome Analysis 2007 Cambridge Studies in Mathematical Biology: Cambridge University Press

[B4] The ENCODE Project consortium ENCODE Nature 2012 489 7414 45 113 10.1038/489045a 22955612

[B5] Chor B Horn D Goldman N Levy Y Massingham T Genomic DNA k-mer spectra: models and modalities Genome Biol 2009 10 R108 10.1186/gb-2009-10-10-r108 19814784 PMC2784323

[B6] Zhou F Olman V Xu Y Barcodes for genomes and applications BMC Bioinf 2008 9 546 10.1186/1471-2105-9-546 PMC2621371 19091119

[B7] Deschavanne PJ Giron A Vilain J Fagot G Genomic Signature: Characterization and Classification of Species Assessed by Chaos Game Representation of Sequences Mol Biol Evol 1999 16 10 1391 1399 10.1093/oxfordjournals.molbev.a026048 10563018

[B8] Hao B Qi J Prokaryote phylogeny without sequence alignment: from avoidance signature to composition distance J Bioinf and Comput Biol 2004 2 1 19 10.1142/S0219720004000442 15272430

[B9] Fofanov Y Luo Y Katili C Wang J Belosludtsev Y Powdrill T Belapurkar C Fofanov V Li TB Chumakov S Pettitt B How independent are the appearances of n-mers in different genomes? Bioinformatics 2008 20 15 2421 2428 15087315 10.1093/bioinformatics/bth266

[B10] Vinga S Almeida Alignment-free sequence comparison - a review Bioinformatics 2003 19 4 513 523 10.1093/bioinformatics/btg005 12611807

[B11] Mantegna RN Buldyrev S Godberger A Havlin S Peng C Simons M Stanley H Linguistic Features of Noncoding DNA Sequences Phys Rev Lett 1994 73 23 3169 3172 10.1103/PhysRevLett.73.3169 10057305

[B12] Hampikian G Andersen T Absent sequences: nullomers and primes Pac Symp Biocomputing 2007 12 355 366 10.1142/9789812772435_0034 17990505

[B13] Haubold B Pierstorff N Möller F Wiehe T Genome comparison without alignment using shortest unique substrings BCM Bioinf 2005 6 123 10.1186/1471-2105-6-123 PMC1166540 15910684

[B14] Ichinose N Yada T Gotoh O Large-scale motif discovery using DNA Gray code and equiprobable oligomers Bioinformatics 2012 28 25 31 10.1093/bioinformatics/btr606 22057160 PMC3244767

[B15] Tai Y Coding-Independent Regulation of the Tumor Suppressor PTEN by Competing Endogenous mRNAs Cell 2011 147 344 357 10.1016/j.cell.2011.09.029 22000013 PMC3235920

[B16] Fici G Mignosi F Restivo A Sciortino M Word assembly through minimal forbidden words Theor Comput Sci 2006 359 214 230 10.1016/j.tcs.2006.03.006

[B17] Cicalese F Erdös P Lipták Z Iliopoulos C, Smyth WF Efficient reconstruction of RC-equivalent strings IWOCA 2010 - LNCS 6460 2011 349 362

[B18] Castellini A Manca V Compri S Tosadori G Bicego M Genome classification by dictionary-based indexes Poster, presented at the Int. Conf. on Pattern Recognition in Bioinformatics (PRIB2011) 2011 TU Delft

[B19] NCBI Genome database [http://www.ncbi.nlm.nih.gov/sites/genome]

[B20] UCSC Genome Bioinformatics website [http://hgdownload.cse.ucsc.edu/downloads.html]

[B21] EMBL-EBI website [http://www.ebi.ac.uk/genomes/]

[B22] QlikView website [http://www.qlikview.com/]

